# Diversity of methanogens in the hindgut of captive white rhinoceroses, *Ceratotherium simum*

**DOI:** 10.1186/1471-2180-13-207

**Published:** 2013-09-12

**Authors:** Yu-heng Luo, André-Denis G Wright, You-long Li, Hua Li, Qi-hong Yang, Ling-juan Luo, Ming-xian Yang

**Affiliations:** 1 Key Laboratory for Animal Disease-Resistance Nutrition of China and Ministry of Education; Institute of Animal Nutrition, Sichuan Agricultural University, 625014, Ya’an, China; 2 Department of Animal Science, University of Vermont, 570 Main Street, Burlington, Vermont 05405, USA; 3 Yunnan Wilde Animals Park, 18 Fengyuan Road, Panlong District, 650218, Kunming, China; 4 Yunnan Natural Forest Center, 80 Jinhun Road, Panlong District, 650224, Kunming, China; 5 Yunnan Business Information Engineering School, Panlong District, 650204, Kunming, China; 6 College of Animal Science and Technology, Sichuan Agricultural University, 625014, Ya’an, China

**Keywords:** White rhinoceros, Methanogen, Gut microbial diversity

## Abstract

**Background:**

The white rhinoceros is on the verge of extinction with less than 20,200 animals remaining in the wild. In order to better protect these endangered animals, it is necessary to better understand their digestive physiology and nutritional requirements. The gut microbiota is nutritionally important for herbivorous animals. However, little is known about the microbial diversity in the gastrointestinal tract (GIT) of the white rhinoceros. Methanogen diversity in the GIT may be host species-specific and, or, function-dependent. To assess methanogen diversity in the hindgut of white rhinoceroses, an archaeal 16S rRNA gene clone library was constructed from pooled PCR products obtained from the feces of seven adult animals.

**Results:**

Sequence analysis of 153 archaeal 16S rRNA sequences revealed 47 unique phylotypes, which were assigned to seven operational taxonomic units (OTUs 1 to 7). Sequences assigned to OTU-7 (64 out of 153 total sequencs – 42%) and OTU-5 (18%, 27/153) had 96.2% and 95.5% identity to *Methanocorpusculum labreanum*, respectively, making *Methanocorpusculum labreanum* the predominant phylotype in these white rhynoceroses. Sequences belonging to OTU-6 (27%, 42/153) were related (97.6%) to *Methanobrevibacter smithii*. Only 4% of the total sequences (6/153) were assigned to *Methanosphaera stadtmanae* (OTU-1). Sequences belonging to OTU-2 (4%, 6/153), OTU-3 (3%, 5/153) and OTU-4 (2%, 3/153) were distantly related (87.5 to 88,4%) to *Methanomassiliicoccus luminyensis* and were considered to be novel species or strains that have yet-to-be cultivated and characterized.

**Conclusion:**

Phylogenetic analysis indicated that the methanogen species in the hindgut of white rhinoceroses were more similar to those in the hindgut of horses. Our findings may help develop studies on improving the digestibility of forage for sustainable management and better health of these endangered animals.

## Background

The white rhinoceros (*Ceratotherium simum*) belongs to the family Rhinocerotidae (order Perrisodactyla) and is the largest of the five species of rhinoceros and the world’s third largest land mammal after the African and Indian elephants. It has a massive body and large head, and its weight ranges from 1,360 to 3,630 kg. White rhinoceroses are herbivore grazers. They spend about half of the day eating grass and are normally found in the savannah and grassland habitats [1]. These large odd-toed ungulates are hindgut colonic fermenters, so they typically have a proportionally longer large intestine than small intestine.

White rhinoceroses are well known for their two horns, which have resulted in many of these animals being killed by poachers for their horns. Now the white rhinoceros is on the International Union for Conservation of Nature and Natural Resources (IUCN) Red List of Threatened Species [2]. The white rhinoceros once roamed much of sub-Saharan Africa, but today is on the near threatened list with less than 20,200 of these animals remaining in the wild [2]. One of the prerequisites to better protect these endangered animal species is to better understand their digestive physiology and nutritional requirements. Given the importance of the gut microbiota in herbivorous animals, little is known about the hindgut microorganisms in the white rhinoceros.

Methanogenic archaea, also called methanogens, exist widely in the GIT of many vertebrates and invertebrates [3]. Methanogens can use a number of different substrates, such as hydrogen, formate, acetate, methanol, and methlyamines, to reduce carbon dioxide to methane during the normal fermentation of feed [4], and studies on ruminants have shown that the production of enteric methane results in loss of gross energy available to the host [5,6]. Methanogens have been isolated from various animals [7,8] and several studies using culture-independent methods, including 16S rRNA gene clone library analysis, have provided some useful data on the diversity and abundance of methanogens in rumen [9-12]. In other hindgut fermenters, such as humans and pigs, the diversity and density of methanogens in the human colon were different among obese and lean, or post-gastric-bypass, individuals [13]. Moreover, the structure of fecal methanogens appears to differ among different pig breeds [14,15]. These studies indicated that methanogen diversity in the GIT may be host species-specific and, or, function-dependent. Therefore, we hypothesize that the methanogens present in the white rhinoceros may have a unique community structure and composition than those from other herbivores, which have been studied to date.

The objectives of the present study are to elucidate the molecular diversity and community structure of methanogens in the hindgut of the white rhinoceroses using 16S rRNA gene clone library analysis.

## Methods

### Sample sources and processing

All animals were legally transported from South Africa into Yunnan Wild Animal Park in China as ornamental animals in July, 2010 under permission of the State Forestry Bureau of China, and were managed according to the guidelines of animal care and use approved by the Chinese Authority.

Seven adult white rhinoceroses (4 males and 3 females), aged from 6 to 8 years old, were selected as experimental animals. Feed consisted of pellets, apple, carrot, fresh forage/alfalfa and alfalfa hay with a ratio as 10:5:10:80:10. The ingredients and proportion of the pellet feed (per 100 kg) were as follows: 30 kg maize, 20 kg soybean meal, 8 kg wheat bran, 8 kg wheat, 5 kg malt root, 3 kg rice bran, 12 kg alfalfa meal, 7 kg oil cake, 1.5 kg yeast extract, 1.5 kg bone meal, 1 kg salt, 1 kg fish meal, 0.1 kg compound vitamins, 0.1 kg lysine, 1.2 kg di-calcium phosphate, 0.1 kg sodium selenite-Vitamin E, 0.7 kg calcium carbonate, 0.1 kg trace element, 0.1 kg zinc sulfate and 0.1 kg copper sulfate. Approximately 10 g of fresh feces were collected from each rhinoceros in August, 2012, and stored on ice in a sterilized 15-ml centrifuge tube until transported to the laboratory (approximately 2 h). Fecal samples were then stored at −20°C until further processing. The collection of the fecal samples and the subsequent analysis was permitted by Yunnan Wild Animal Park and the State Forestry Bureau of China.

### DNA extraction, PCR amplification and clone library construction

Nucleic acids were extracted from 0.5 g of feces using the bead-beating method described by Zoetendal et al. [16], and DNA samples were purified with a PCR Clean-Up system (Promega, Madison, USA) and stored at −20°C.

Methanogen specific primers Met86F and Met1340R [17] were used to amplify archaeal 16S rRNA genes. The amplification was initiated with a denaturation at 94°C for 3 min, followed by 40 cycles of 94°C for 30 s, 58°C for 30 s and 72°C for 90 s, and a last extension at 72°C for 10 min. The PCR reaction mixture (50 μl) consisted of 200 nM of each primer, approximately 0.35 μg of template DNA, 1 × Taq reaction buffer, 200 μM of each dNTP, 2 mM of MgCl_2_ and four units of *Taq* DNA polymerase. The amplicons were purified using a PCR Clean-Up system (Promega, Madison,USA).

A 16S rRNA gene clone library was constructed using equal quantities of purified pooled PCR products from each animal, that had been cloned into the pGEM-T Easy vector and transformed into *Escherichia coli* TOP10 (Promega, Madison,USA). A total of 160 transformed clones with correct sized inserts were selected and confirmed by sequence analysis (Invitrogen, Shanghai, China).

### Estimation of archaeal diversity and phylogenetic analysis

Sequences were checked for chimeras using the chimera detection program BELLERPHON as part of the software package MOTHUR (ver 1.23.1). Based on a species-level sequence identity criterion of 98% [18], MOTHUR was used to assign the 16S rRNA gene sequences to operational taxonomic units (OTUs). The sampling effort in the library for species-level OTUs was evaluated by calculating the coverage (C) according to the equation C = 1 - (n/N), where n is the number of OTUs represented by a single clone and N is the total number of clones analyzed in the library [19]. GenBank’s Basic Local Alignment Search Tool (BLAST) [20] was used to presumptively identify the nearest validly described neighbor of each methanogen sequence. Lastly, a neighbor-joining tree was constructed using the phylogenetic software PHYLIP (ver 3.69) with 1,000 bootstrap resamplings of the dataset [21]. The nucleotide sequences reported in this paper have been deposited in the GenBank database under accession numbers JX833566 to JX833612.

## Results

**Figure 1:**
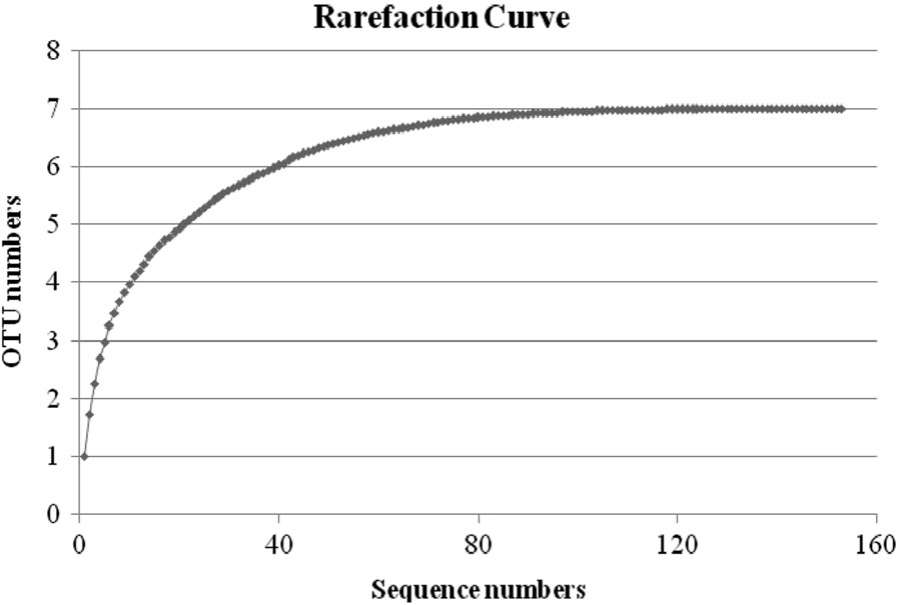
**Phylogenetic relationship of archaeal 16S rRNA gene sequences retrieved from fecal samples of white rhinoceroses.** Evolutionary distances were calculated using the Neighbor-Joining method. The tree was bootstrap resampled 1000 times.

**Figure 2:**
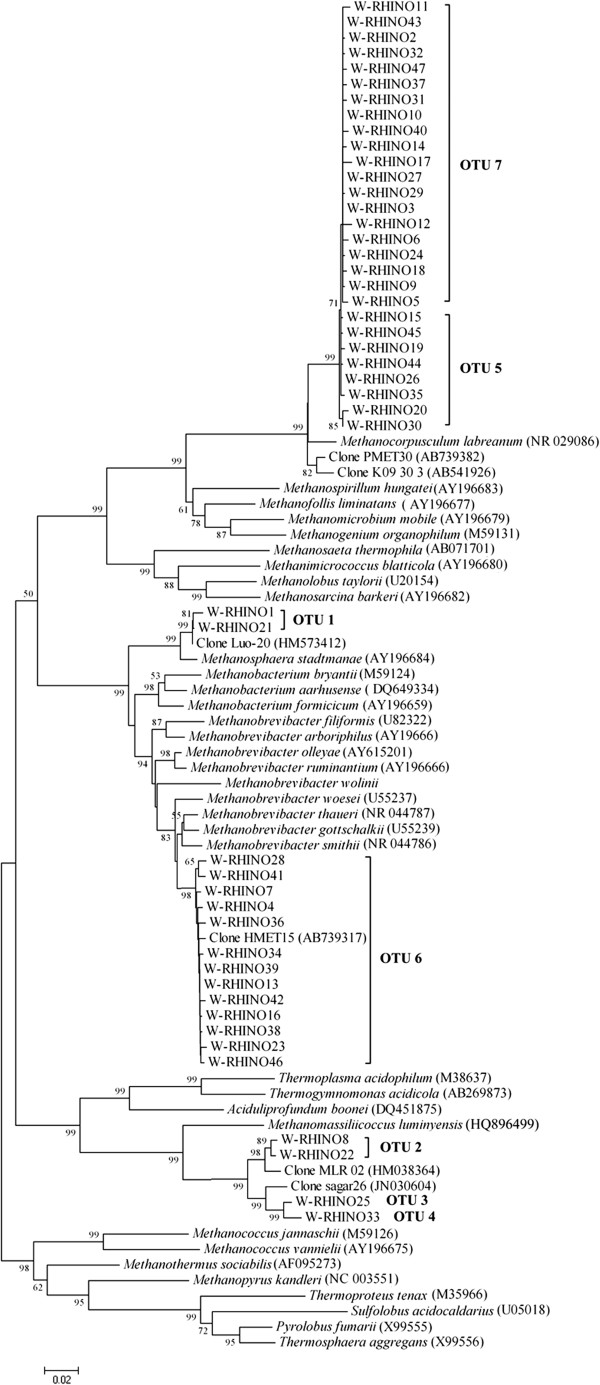
Rarefaction curve of the archaeal 16S rRNA clone library obtained from hindgut of the white rhinoceroses.

A total of 153 non-chimeric 16S rRNA gene sequences were obtained from fecal samples of seven white rhinoceroses. Examination of the 153 sequences revealed 47 different phylotypes (Figure 1), which were assigned to 7 OTUs based on a 98% sequence identity criterion (Table 1). The coverage of the clone library was 95.4%, indicating the library was well sampled (Figure 2). The CHAO 1 OTU estimate was 7, and the Shannon Index was 1.47 ± 0.13. Six sequences (4%) were assigned to OTU-1 and had 96.6% identity to *Methanosphaera stadtmanae* (Table 1). OTU-2 (6 sequences), OTU-3 (5 sequences) and OTU-4 (3 sequences) were distantly related to *Methanomassiliicoccus luminyensis* with sequences ranging from 87.5% to 88.4%. OTU-5 (27 sequences) and OTU-7 (64 sequences) were related to *Methanocorpusculum labreanum* with sequence identities of 96.2% and 95.5%, respectively. Forty-two sequences (27%) were assigned to OTU-6 and had 97.3% to 97.6% sequence identity to *Methanobrevibacter smithii*.

**Table 1:** Operational taxonomic units (OTUs) of archaeal 16S rRNA gene sequences from feces of white rhinoceroses

**OTU**	**phylotype**	**No. of sequences**	Nearest valid taxon*	**% Sequence**	**Nearest uncharacterized**	**% Sequence**
				**identity**	**clone**	**identity**
1	W-Rhino1	2	*Methanosphaera stadtmanae*	96.3	HM573412	99.4
1	W-Rhino21	4	*Methanosphaera stadtmanae*	96.6	HM573412	99.8
2	W-Rhino8	4	*Methanomassiliicoccus luminyensis*	88.1	HM038364	98.6
2	W-Rhino22	2	*Methanomassiliicoccus luminyensis*	88.4	HM038364	98.6
3	W-Rhino25	5	*Methanomassiliicoccus luminyensis*	87.8	JN030604	95.9
4	W-Rhino33	3	*Methanomassiliicoccus luminyensis*	87.5	JN030608	95.7
5	W-Rhino15	6	*Methanocorpusculum labreanum*	95.5	AB739382	95.9
5	W-Rhino19	2	*Methanocorpusculum labreanum*	95.1	AB739382	95.7
5	W-Rhino20	5	*Methanocorpusculum labreanum*	95.1	AB739382	96.0
5	W-Rhino26	3	*Methanocorpusculum labreanum*	95.5	AB739382	96.3
5	W-Rhino30	2	*Methanocorpusculum labreanum*	95.1	AB739382	96.0
5	W-Rhino35	6	*Methanocorpusculum labreanum*	95.3	AB739382	95.8
5	W-Rhino44	1	*Methanocorpusculum labreanum*	95.4	AB739382	95.9
5	W-Rhino45	2	*Methanocorpusculum labreanum*	95.4	AB739382	95.9
6	W-Rhino4	3	*Methanobrevibacter smithii*	97.3	AB739317	98.9
6	W-Rhino7	5	*Methanobrevibacter smithii*	97.5	AB739317	99.4
6	W-Rhino13	1	*Methanobrevibacter smithii*	97.6	AB739317	99.6
6	W-Rhino16	7	*Methanobrevibacter smithii*	97.5	AB739317	99.5
6	W-Rhino23	11	*Methanobrevibacter smithii*	97.5	AB739317	99.4
6	W-Rhino28	4	*Methanobrevibacter smithii*	97	AB739317	98.7
6	W-Rhino34	4	*Methanobrevibacter smithii*	97.5	AB739317	99.5
6	W-Rhino36	1	*Methanobrevibacter smithii*	97.4	AB739317	99.4
6	W-Rhino38	1	*Methanobrevibacter smithii*	97.5	AB739317	99.4
6	W-Rhino39	1	*Methanobrevibacter smithii*	97.6	AB739317	99.6
6	W-Rhino41	2	*Methanobrevibacter smithii*	97.4	AB739317	99.3
6	W-Rhino42	1	*Methanobrevibacter smithii*	97.4	AB739317	99.4
6	W-Rhino46	1	*Methanobrevibacter smithii*	97.5	AB739317	99.4
7	W-Rhino2	3	*Methanocorpusculum labreanum*	95.4	AB739382	96.2
7	W-Rhino3	1	*Methanocorpusculum labreanum*	95.4	AB739382	96.2
7	W-Rhino5	5	*Methanocorpusculum labreanum*	95.2	AB739382	96.2
7	W-Rhino6	9	*Methanocorpusculum labreanum*	95.2	AB739382	95.7
7	W-Rhino9	4	*Methanocorpusculum labreanum*	95.4	AB739382	96.2
7	W-Rhino10	1	*Methanocorpusculum labreanum*	95.4	AB541926	96.0
7	W-Rhino11	3	*Methanocorpusculum labreanum*	95.1	AB541926	95.8
7	W-Rhino12	7	*Methanocorpusculum labreanum*	95.1	AB541926	95.6
7	W-Rhino14	2	*Methanocorpusculum labreanum*	95.2	AB541926	95.8
7	W-Rhino17	2	*Methanocorpusculum labreanum*	95.1	AB739382	95.9
7	W-Rhino18	1	*Methanocorpusculum labreanum*	95.3	AB739382	96.1
7	W-Rhino24	2	*Methanocorpusculum labreanum*	95.4	AB739382	96.2
7	W-Rhino27	1	*Methanocorpusculum labreanum*	95.6	AB541926	96.0
7	W-Rhino29	7	*Methanocorpusculum labreanum*	95.3	AB739382	96.1
7	W-Rhino31	1	*Methanocorpusculum labreanum*	95.3	AB739382	96.1
7	W-Rhino32	2	*Methanocorpusculum labreanum*	96.2	AB739400	96.9
7	W-Rhino37	5	*Methanocorpusculum labreanum*	95.3	AB739382	96.1
7	W-Rhino40	1	*Methanocorpusculum labreanum*	95.2	AB739382	96.0
7	W-Rhino43	3	*Methanocorpusculum labreanum*	95.4	AB739382	96.2
7	W-Rhino47	4	*Methanocorpusculum labreanum*	95.2	AB739382	96.0
Totals		153				

*Nearest valid taxon with the same name means the same strain.

At the phylotype level, W-Rhino1 and W-Rhyno21 (both assigned to OTU-1) were closely related to an uncharacterized archaeal clone from pig feces (99.4% and 99.8% identities, respectively) [14] (Table 1, Figure 1). The two phylotypes belonging to OTU-2 had 98.6% identity to an uncultured clone from bovine rumen [22] (Table 1, Figure 1). Two sequences were related to two methanogen clones (JN030604 and JN030608) from continental shelf of India with 96.0% and 95.7% identity, respectively (Table 1, Figure 1). Five sequences assigned to OTU-7 showed genus-level (95.6% to 96%) sequence identity to an uncharacterized clone from cattle manure [23], while the remaining phylotypes that were assigned to OTU-7 were related to a methanogen clone from the hindgut of the pony (AB739382) with 95.7% to 96.9% identities (Table 1, Figure 1). All phylotypes assigned to OTU-5 also showed genus-level (95.7 to 96.3%) sequence identity to a clone from the hindgut of the pony (AB739382) (Table 1, Figure 1). The clone library OTU coverage rate was 95.4%, indicating that the library was very well sampled for the diversity it contained.

**Figure 3:**
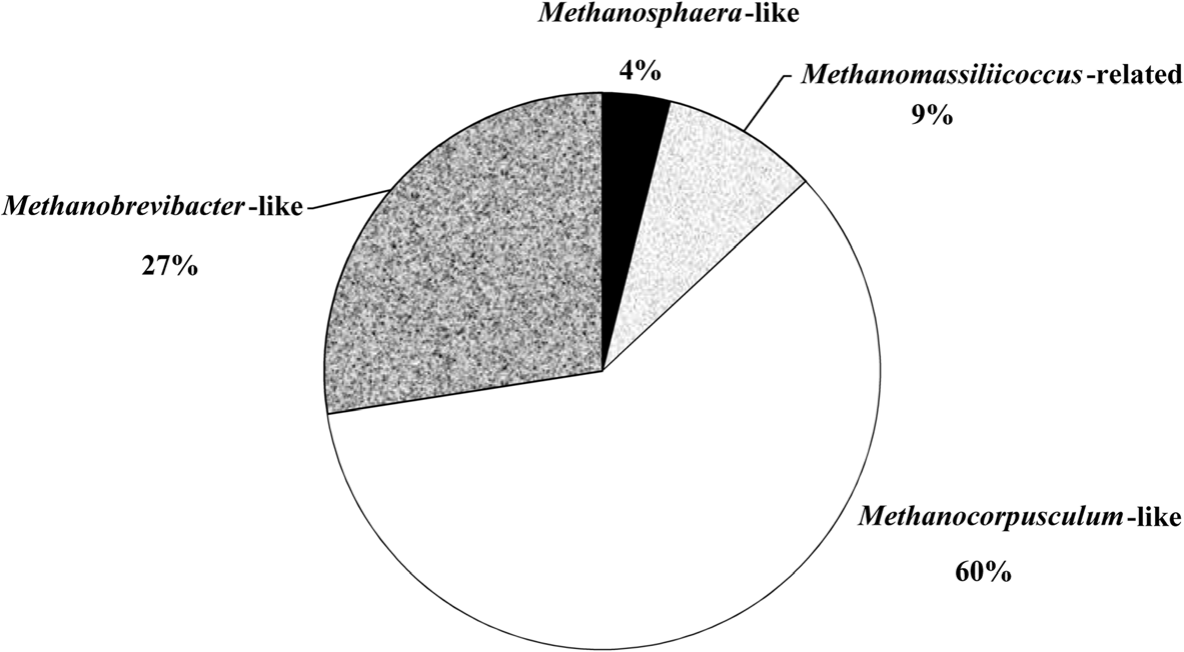
**Pie chart representation of methanogen 16S rRNA gene clone distributions in feces of white rhinoceroses.***Methanocorpusculum*-like sequences represented the majority in the library (60%), followed by *Methanobrevibacter-*like (27%), *Methanomassiliicoccus-*related (9%) and *Methanosphaera-*like (4%).

Phylogenetic analysis indicated that all 47 phylotypes (i.e., 153 sequences) belonged to four monophyletic groups (Figures 1 and 3). Phylotypes assigned to OTU-5 and OTU-7 grouped together on a branch as the sister group to *Methanocorpusculum labreanum* with very strong bootstrap support (99%), OTU-1 phylotypes grouped within the genus *Methanosphaera*, and phylotypes assigned to OTU-6 grouped together on a branch with several species belonging to the genus *Methanobrevibacter*. The remaining phylotypes grouped together with other uncultivated methanogens belonging to a recently proposed seventh order of methanogenic archaea, the *Methanoplasmatales*[24].

## Discussion

To the best of our knowledge, the current study is the first to report methanogens closely related to *Methanocorpusculum labreanum*[25] as the predominant phylotype in the gastrointestinal tract of animals. This is in contrast to many other studies, where *Methanobrevibacter* species were the dominant methanogen phylotypes in other herbivores worldwide [26-30]. In the present study, approximately 60% of the 153 16S rRNA gene sequences obtained from the feces of white rhinoceroses was related to the genus *Methanocorpusculum*. However, it is important to note that the use of a pooled sample makes it impossible to know if these methanogens were prevalent in all seven animals. In contrast, the proportion of the sequences assigned to the genus *Methanobrevibacter* was only 27%.

Studies on ruminants [10] and on monogastric animals, such as pigs and gnotobiotic mice [14,31], have indicated that *Methanobrevibacter smithii* affects the efficiency of digestion of dietary polysaccharides, whereas most strains of *Methanocorpusculum labreanum* have been isolated from sediments, anaerobic digesters, waste water [32,33], and the hindgut of termites [34,35]. *Methanocorpusculum labreanum* also requires acetate as a carbon source and has additional complex nutritional requirements [36]. Termites, horses and very large herbivores such as rhinoceroses and elephants are typical hindgut fermenters [37]. The common distribution of *Methanocorpusculum labreanum* in the hindgut of termites and rhinoceroses may likely be due to the digestive physiology of the hindgut and may play an unusual function for digestion of dietary fibers.

Facey et al. [38] found that *Methanosphaera stadtmanae,* a methanol utilizer, was the predominant methanogen in the gastrointestinal tract of orangutans. The researchers suggested that the high prevalence of *Methanosphaera stadtmanae* may likely due to the increased availability of methanol from the highly frugivorous diet of the orangutans. *Methanosphaera* stadtmanae was also found in the current study, but was represented in only 4% of the total sequences. Like other species of *Methanobrevibacter*, *Methanocorpusculum labreanum* also produces methane from H_2_-CO_2_, or formate, but not from methanol and methylanmines [24]. Thus, we inferred that the low representation of *Methanosphaera* stadtmanae may be due to the predominant presence of *Methanocorpusculum labreanum*, or because of the small quantity of methanol produced by the fermentation of plant material in the hindgut of the white rhinoceroses, which needs to be further studied.

Based on calculations derived from *in vitro* studies and domestic ruminants, the growth of gut methanogens has been postulated to be a limiting factor in large herbivore digestive physiology [39]. For example, the relatively fast passage rates in elephants, the largest extant terrestrial mammal, have been interpreted in part as a counter-measure against the danger of disproportional methanogen growth [37]. However, for some smaller mammalian or reptilian herbivores, the food particle retention times surpass the 4-day threshold postulated by Van Soest (1994). In these species, the fermentation products are better absorbed and not available as substrate for slow-growing methanogens. Therefore, we speculate that the particular species of methanogens found in the hindgut of the white rhinoceros may be well suited in these large herbivores and play an unique role during the fermentation of the plant materials. Further studies on the function of these methanogen species are needed.

In the present study, the majority of methanogen sequences showed a closer relationship to uncharacterized clones in the equine hindgut. W-Rhino8 (assigned to OTU-2) was closely related to a methanogenic clone from the hindgut of the horse. All phylotypes belonging to OTU-5 and 15 phylotypes from OTU-7 were also related (96.9%) to an uncultured archaeal clone from the hindgut of a pony. In a previous study, the horse was identified as an appropriate model when designing diets for captive animals such as large hindgut fermenters, elephants or rhinoceroses [40]. It is also been reported that the Indian rhinoceros resembles the domestic horse in most digestive characteristics, despite the immense body size difference between the species [1]. Interestingly, rhinoceroses and horses are both odd-toed ungulates belonging to the order Perissodactyla. Thus, the closer phylogenetic relationship of methanogenic species between rhinoceroses and horses may be associated with the common characteristics of their GIT (i.e. microbial habitat).

Our library also uncovered some unidentified archaeal sequences belonging to OTU-2, OTU-3 and OTU-4. The sequences were only 87.8% to 88.4% similar to *Methanomassiliicoccus luminyensis*, a new methanogen recently isolated from human stool [41] and belonging to the newly proposed order Methanoplasmatales [24].

## Conclusions

In conclusion, the white rhinoceros harbors a unique fecal community of methanogens distinct from other animals, but with more similarity to horses and ponies. *Methanocorpusculum labreanum* represents the predominant (60%) methanogenic species in the hindgut of the white rhinoceros. A number of novel methanogen sequences were also found, but their functional role in the digestion and health of the white rhinoceros awaits further investigation.

### Availability of supporting data

The data sets supporting the results of this article are included within the article.

## Competing interests

The authors declare that they have no competing interests.

## Authors’ contributions

YL designed the study, carried out the sequence alignment and drafted the manuscript. ADGW participated in the sequence alignment and performed the statistical analysis. YL participated in the design of the study. HL participated in the sequence alignment. QY participated in the design of the study. LL and MY helped to draft the manuscript. All authors read and approved the final manuscript.
